# A Conformational Shift in the Dissociated Cholera Toxin A1 Subunit Prevents Reassembly of the Cholera Holotoxin

**DOI:** 10.3390/toxins7072674

**Published:** 2015-07-20

**Authors:** Michael Taylor, David Curtis, Ken Teter

**Affiliations:** Burnett School of Biomedical Sciences, College of Medicine, University of Central Florida, 12722 Research Parkway, Orlando, FL 32826, USA; E-Mail: serenity0891@gmail.com

**Keywords:** cholera toxin, endoplasmic reticulum, protein disulfide isomerase, surface plasmon resonance, toxin assembly, toxin instability

## Abstract

Cholera toxin (CT) consists of a catalytic A1 subunit, an A2 linker, and a homopentameric cell-binding B subunit. The intact holotoxin moves by vesicle carriers from the cell surface to the endoplasmic reticulum (ER) where CTA1 is released from the rest of the toxin. The dissociated CTA1 subunit then shifts to an unfolded conformation, which triggers its export to the cytosol by a process involving the quality control system of ER-associated degradation (ERAD). We hypothesized that the unfolding of dissociated CTA1 would prevent its non-productive reassociation with CTA2/CTB_5_. To test this prediction, we monitored the real-time reassociation of CTA1 with CTA2/CTB_5_ by surface plasmon resonance. Folded but not disordered CTA1 could interact with CTA2/CTB_5_ to form a stable, functional holotoxin. Our data, thus, identified another role for the intrinsic instability of the isolated CTA1 polypeptide in host-toxin interactions: in addition to activating the ERAD translocation mechanism, the spontaneous unfolding of free CTA1 at 37 °C prevents the non-productive reassembly of a CT holotoxin in the ER.

## 1. Introduction

**Figure 1:**
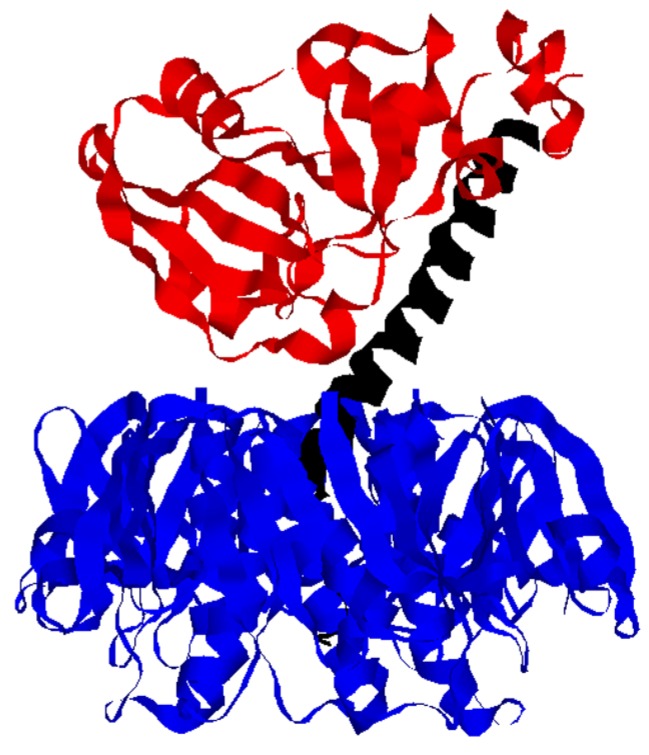
CT Structure. The catalytic CTA1 subunit (**red**) is anchored to the CTA2 subunit (**black**) by numerous non-covalent interactions and a single disulfide bond between the *C*-terminus of CTA1 and *N*-terminus of CTA2. The A2 linker extends into the central pore of the ring-like B homopentamer (**blue**) and thus maintains extensive non-covalent contacts with CTB. Separation of CTA1 from CTA2/CTB_5_ is required for the ER-to-cytosol translocation of CTA1. The image was derived from PBD 1S5F [7].

Cholera toxin (CT) is an AB_5_ protein toxin that consists of a catalytic A subunit and a homopentameric, cell-binding B subunit [1,2] (Figure 1). The two components are organized from A and B monomers into an intact AB_5_ holotoxin in the bacterial periplasm [3]. Post-translational nicking of the A subunit generates an A1/A2 heterodimer with a disulfide bridge spanning the cleavage site [4,5]. CTA1 is an ADP-ribosyltransferase that modifies and activates the stimulatory α subunit of the heterotrimeric G protein (Gsα) inside the host cell. CTA2 is an α-helical peptide that, in addition to the CTA1/CTA2 disulfide bond, maintains numerous non-covalent interactions with CTA1. CTA2 also forms a stable, non-covalent complex with CTB through the positioning of its *C*-terminus within the central pore of the B pentamer. CTA2, thus, acts as a linker between the catalytic and cell-binding components of CT. In addition, a tetrapeptide KDEL motif at the *C*-terminus of CTA2 enhances the accumulation of toxin within the ER [6].

To reach its cytosolic Gsα target, CT first binds to GM1 gangliosides on the plasma membrane of a target cell. The toxin is then delivered from the cell surface to the endoplasmic reticulum (ER) through a series of vesicular trafficking events collectively termed retrograde transport [8]. As it travels to the ER, CTA1 is held in a stable conformation by its disulfide bridge to CTA2 and its assembly in the CT holotoxin [9,10,11]. The CTA1/CTA2 disulfide bond is reduced at the resident redox state of the ER [12], but this alone is not sufficient to remove CTA1 from the rest of the toxin [5,13]: an additional contribution from protein disulfide isomerase (PDI) is required to displace CTA1 from CTA2/CTB_5_ [14,15,16]. PDI does not directly affect the secondary structure of CTA1, and the conformation of free CTA1 is nearly identical to the conformation of PDI-exposed CTA1 [16]. However, the PDI-mediated displacement of reduced CTA1 from CTA2/CTB_5_ releases the structural constraints on CTA1 unfolding and allows the toxin to spontaneously shift to a disordered state at physiological temperature [9,16]. The spontaneous unfolding of dissociated CTA1 displaces its PDI binding partner [16] and places the toxin subunit in a translocation-competent conformation [17,18,19] for passage into the cytosol through one or more protein-conducting pores in the ER membrane [20,21,22,23,24]. Translocation is facilitated by ER-associated degradation (ERAD), a quality control system that recognizes the disordered CTA1 subunit as a substrate for ER-to-cytosol export [17,18,19,25,26]. The arginine-over-lysine amino acid bias in CTA1 allows the toxin to avoid the usual ubiquitin-dependent proteasomal degradation of ERAD substrates [9,27,28]. Yet free CTA1 is in a disordered conformation at 37 °C [9] and consequently lacks enzymatic activity [29]. An interaction with ADP-ribosylation factors and other host factors is therefore required for cytosolic CTA1 to regain a folded, active conformation at physiological temperature [30,31,32,33,34].

PDI does not remain bound to CTA1 after holotoxin disassembly [16]. As such, the free CTA1 subunit could potentially reassociate with CTA2/CTB_5_ in the ER. This phenomenon has been reported for ricin, another AB-type toxin: plasmid-borne expression of ricin B chain in the ER of ricin-intoxicated cells resulted in the capture of free, ER-localized ricin A chain and consequently protected the transfected cells from ricin challenge [35]. The individual ricin A and B subunits can also form an AB holotoxin when combined *in vitro* [36]. We hypothesized the thermal unfolding of the dissociated, free CTA1 subunit would place it in a conformation that prevents non-productive reassembly of a cholera holotoxin in the ER. This prediction was tested by monitoring the assembly of a cholera holotoxin with surface plasmon resonance (SPR). We found that CTA1 could associate with CTA2/CTB_5_ when held in a folded conformation, but the disordered 37 °C conformation of CTA1 could not interact with CTA2/CTB_5_. The reconstituted holotoxin was a stable complex that could be disassembled by PDI and could intoxicate cultured CHO cells. Our data have thus defined a new function for the intrinsic instability of the isolated CTA1 polypeptide: in addition to activating the ERAD translocation mechanism, the spontaneous unfolding of dissociated CTA1 at physiological temperature prevents the non-productive reassembly of a CT holotoxin in the ER.

## 2. Results and Discussion

**Figure 2:**
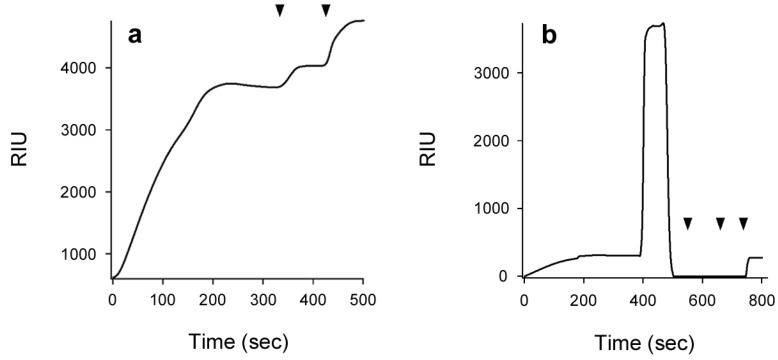
CTA1 re-association with CTA2/CTB_5_. (**a**) CTA1 was added to a CTA2/CTB_5_ SPR sensor at 21 °C, removed from the perfusion buffer after 200 s, and replaced with sequential perfusions of anti-CTA1 and anti-CTB antibodies as indicated by the arrowheads; (**b**) A baseline measurement corresponding to the mass of the CTA2/CTB_5_ complex established the 0 RIU signal. The time course was then initiated with perfusion of CTA1 over the CTA2/CTB_5_ sensor at 21 °C. CTA1 was removed from the perfusion buffer after 200 s and replaced with reduced PDI at 400 s. PDI was removed from the perfusion buffer at 550 s and replaced with sequential perfusions of anti-PDI, anti-CTA1, and anti-CTB antibodies as indicated by the arrowheads.

Using SPR, Ampapathi *et al.* [30] calculated a *K*_D_ of 53 nM for the interaction between His-tagged CTA1 and sensor-bound CTA2. This experiment was performed at 21 °C, a temperature that maintains CTA1 in a folded conformation [9]. We therefore predicted that, at 21 °C, CTA1 would re-associate with CTA2/CTB_5_. To examine the interaction between CTA1 and the CTA2/CTB_5_ complex, we appended CTA2/CTB_5_ to a GM1-coated SPR sensor slide. For SPR, a protein of interest is perfused over a sensor slide that is coated with the second protein of interest. When the two proteins interact, the protein in the perfusion buffer is captured on the slide. This increases the mass on the slide, which produces a change in the resonance angle of the reflected light that is recorded as a refractive index unit (RIU) [37,38,39]. An increased RIU signal was recorded when CTA1 was perfused over the CTA2/CTB_5_ slide at 21 °C (Figure 2a). Only a minimal loss of signal occurred when CTA1 was removed from the perfusion buffer, which indicated CTA1 was tightly bound to the CTA2/CTB_5_ complex. The positive signals generated by sequential perfusions of anti-CTA1 monoclonal and anti-CTB polyclonal antibodies over the CTA1-treated slide confirmed that CTA1 had been captured by the sensor-bound CTA2/CTB_5_ complex. RIU signals from our antibody controls were weaker than the signal obtained from CTA1 because sub-saturating antibody concentrations were used for a number of reasons: conservation of the antibodies, low monoclonal antibody concentration in the collected culture medium, and scaling purposes (the pentameric nature of the B subunit contributes to a large RIU signal when higher concentrations of the anti-CTB polyclonal antibody are applied to the sensor). Collectively, these experiments demonstrated that folded CTA1 can re-associate with CTA2/CTB_5_.

Previous work has shown PDI will displace CTA1 from the CT holotoxin under reducing conditions [14,15,16]. This effect was also observed with our reconstituted holotoxin (Figure 2b). When reduced PDI was added to the perfusion buffer after holotoxin reassembly, an initial rise in RIU was followed by a return to the baseline value reflecting the mass of the GM1-bound CTA2/CTB_5_ complex. This occurred despite the continued presence of reduced PDI in the perfusion buffer. Thus, PDI binding to the reconstituted holotoxin resulted in the displacement of CTA1 from the rest of the toxin. Both PDI and CTA1 were swept away in the perfusion buffer and lost from the sensor, so the RIU signal returned to its baseline value. We confirmed this interpretation with sequential perfusions of anti-PDI, anti-CTA1, and anti-CTB antibodies over the sensor slide. A positive signal was only obtained with the anti-CTB antibody, which demonstrated that both PDI and CTA1 had been removed from the remaining CTA2/CTB_5_ complex. The similar interplay between PDI and either CT or reconstituted CT suggested the orientation of CTA1 in our reconstituted CT was similar to its orientation in the pathogen-produced toxin. Thus, the binding/association of CTA1 with CTA2/CTB_5_ at 21 °C appeared to result in reassembly of the CT holotoxin.

**Figure 3:**
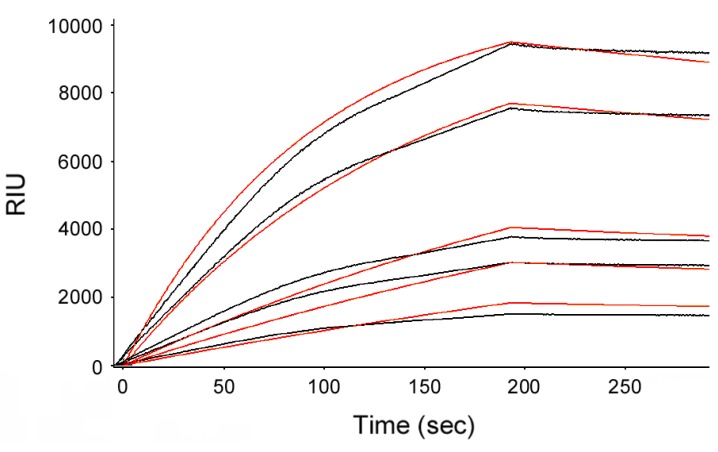
Affinity of CTA1 for CTA2/CTB_5_. A baseline measurement corresponding to the mass of the CTA2/CTB_5_ complex established the 0 RIU signal. The time course was then initiated with perfusion of CTA1 over the CTA2/CTB_5_ sensor at 21 °C. Five concentrations of CTA1 were used in separate perfusions: 185, 100, 50, 31, and 12.5 nM. CTA1 was removed from the perfusion buffer after 200 s. With the BioLogic Scrubber 2 software (Campbell, Australia), the collective data from three separate experiments were used to calculate an on rate of 521 ± 43 M^−1^·s^−1^ for the association of CTA1 with CTA2/CTB_5_. Black lines represent the experimental SPR signals from a single experiment, while orange lines represent the quantified fit (*R*^2^ = 0.9424) from all three experiments.

Additional SPR experiments estimated the affinity between CTA1 and CTA2/CTB_5_ at 21 °C (Figure 3). We recorded a relatively slow on rate of 521 ± 43 M^−1^·s^−1^ (*n* = 3), but CTA1 did not dissociate from CTA2/CTB_5_ after holotoxin reassembly. As such, we could not calculate an off rate or *K*_D_ value for the binding of CTA1 to CTA2/CTB_5_. In comparison to the interaction between CTA1 and CTA2/CTB_5_ (Figure 3), the interaction between CTA1 and free CTA2 exhibited much faster on and off rates [30]. The CTB pentamer thus appears to impede the initial binding of CTA1 to CTA2 but subsequently stabilizes the A1/A2 interface to generate an intact holotoxin. Since β-mercaptoethanol was present in the perfusion buffer, the stability of reconstituted CT did not require a disulfide bridge between CTA1 and CTA2.

**Figure 4:**
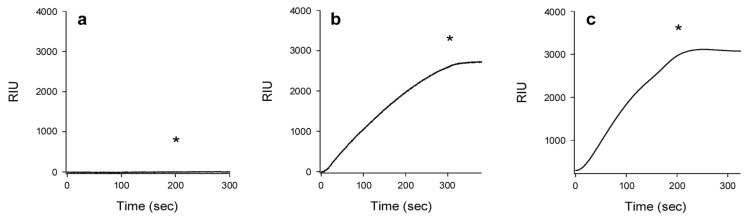
Conformation-dependent association of CTA1 with CTA2/CTB_5_. A baseline measurement corresponding to the mass of the CTA2/CTB_5_ complex established the 0 RIU signal. The time course was then initiated with perfusion of CTA1 over the CTA2/CTB_5_ sensor at (**a**) 37 °C; (**b**) 37 °C with 10% glycerol in the perfusion buffer; or (**c**) 37 °C with 100 μM PBA in the perfusion buffer. An asterisk denotes when CTA1 was removed from the perfusion buffer.

The free CTA1 polypeptide has a highly disordered tertiary structure and a partially perturbed secondary structure at the physiological temperature of 37 °C [9]. We found the disordered, 37 °C conformation of CTA1 could not bind to CTA2/CTB_5_ (Figure 4a). The lack of interaction was conformation-dependent rather than temperature-dependent, as evidenced by the reconstitution of a holotoxin at 37 °C in perfusion buffer containing 10% glycerol (Figure 4b) or 100 μM 4-phenylbutyric acid (PBA) (Figure 4c). Both reagents act as chemical chaperones that maintain CTA1 in a folded conformation at physiological temperature [17,18]. The ability of CTA1 to associate with CTA2/CTB_5_ thus depends on its structural state: folded CTA1 will bind to CTA2/CTB_5_, whereas disordered CTA1 will not.

**Figure 5:**
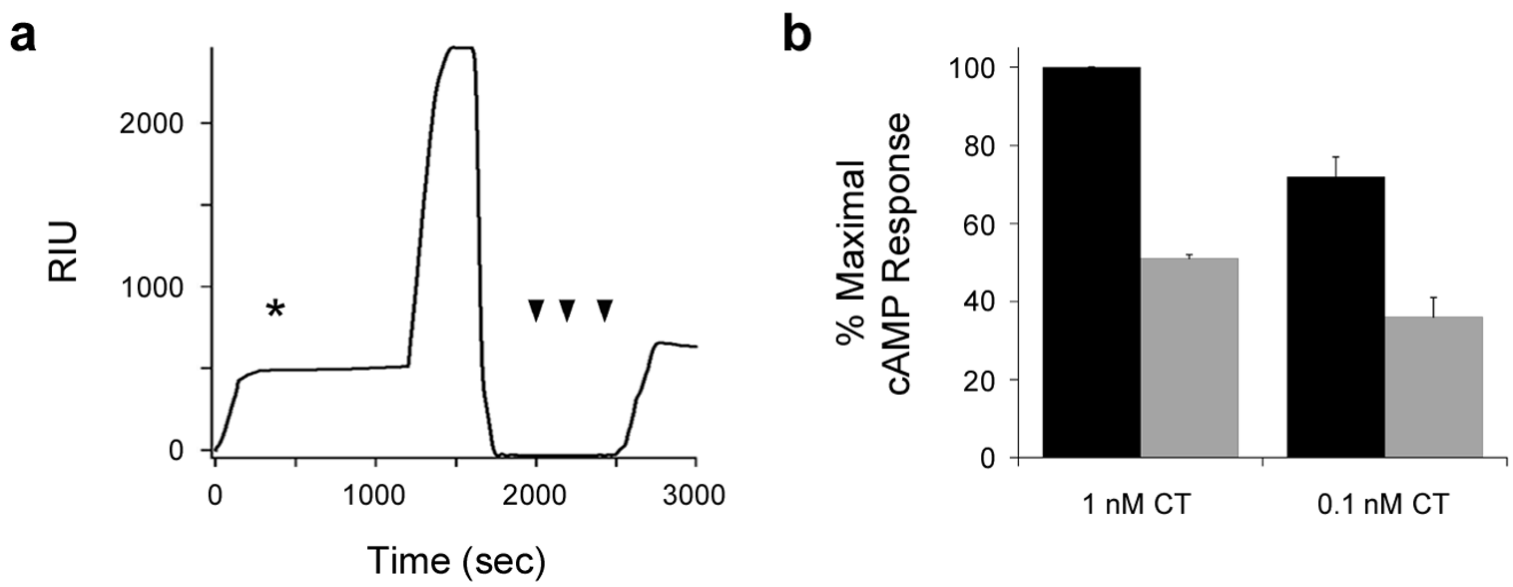
CTA1 re-association with CTA2/CTB_5_ generates a stable, functional holotoxin. (**a**) A baseline measurement corresponding to the mass of the CTA2/CTB_5_ complex established the 0 RIU signal. The time course was then initiated with perfusion of CTA1 over the CTA2/CTB_5_ sensor at 37 °C in pH 6.5 buffer. CTA1 was removed from the perfusion buffer after 200 s and replaced with pH 7.2 buffer as denoted by the asterisk. PDI was subsequently added to the perfusion buffer at 1200 s and removed from the perfusion buffer at 2000 s. Sequential perfusions of anti-PDI, anti-CTA1, and anti-KDEL antibodies are indicated by the arrowheads; (**b**) CHO cells were challenged with the stated concentrations of pathogen-produced CT holotoxin (black bars) or re-assembled CT holotoxin (grey bars). After 2 h at 37 °C, cAMP levels were quantified. Background-subtracted results were expressed as percentages of the maximal cAMP value obtained from cells exposed to 1 nM of pathogen-produced holotoxin. Data represent the averages ± ranges of two independent experiments with triplicate samples.

Toxin-bound CTA1 remained associated with CTA2/CTB_5_ after its removal from the perfusion buffer (Figure 2, Figure 3 and Figure 4). The stability of the reconstituted holotoxin was further demonstrated by subjecting it to a condition that promotes the unfolding of CTA1. We reconstituted a CT holotoxin by perfusing CTA1 over a CTA2/CTB_5_-coated SPR slide at 37 °C in pH 6.5 buffer. Like glycerol and PBA, mildly acidic pH maintains CTA1 in a folded conformation at physiological temperature [19]. A transition from pH 6.5 to pH 7.4 at 37 °C will trigger the unfolding of CTA1 [16], but this pH shift did not result in the displacement of CTA1 from its non-covalent association with CTA2/CTB_5_ (Figure 5a). The re-association of folded CTA1 with CTA2/CTB_5_ at 37 °C and pH 6.5 thus produced a stable complex that prevented the unfolding and/or release of holotoxin-associated CTA1 at 37 °C and pH 7.2. However, reduced PDI could still displace CTA1 from the re-associated holotoxin (Figure 5a).

The re-association of CTA1 with CTA2/CTB_5_ produced a functional holotoxin. Equimolar amounts of CTA1 and CTA2/CTB_5_ were combined at 4 °C for 2 h. The mixture was then applied to CHO cells for 2 h at 37 °C. As shown in Figure 5b, the reconstituted holotoxin elicited a dose-dependent cAMP response from intoxicated cells. The cellular response to reconstituted CT was weaker than the response to pathogen-produced toxin, which was likely due to the inefficiency of toxin re-assembly: the amount of reconstituted toxin applied to cultured cells reflects the initial molar quantities of CTA1 and CTA2/CTB_5_ rather than the final concentration of reconstituted holotoxin. Still, 0.1 nM of the reconstituted CT produced a robust cAMP response that was 10-fold greater than the resting levels of cAMP (150 fmol/well) from unintoxicated control cells. Folded CTA1 can thus re-associate with the CTA2/CTB_5_ complex to form a stable, functional holotoxin. This can accordingly be viewed as an assembly event. If holotoxin reassembly occurred in the ER, however, the A1 subunit would be prevented from reaching the cytosol and its G protein target. The spontaneous unfolding of dissociated CTA1 at physiological temperature thus appears to enhance the efficiency of intoxication by preventing recurring cycles of holotoxin disassembly and assembly in the ER.

## 3. Experimental Section

### 3.1. Isolation of CTA1 from CTA

To separate the 21 kDa CTA1 subunit from the 5 kDa CTA2 subunit, 5 μg of the disulfide-linked CTA1/CTA2 heterodimer (CTA; Sigma-Aldrich, St. Louis, MO, USA) placed in 0.5 mL of 10 mM sodium borate buffer (pH 7.1) containing 10 mM β-mercaptoethanol and 150 mM NaCl was dialyzed against the same buffer for 1 h at 4 °C in a 10 kDa MWCO Slide-a-Lyzer cassette (Life Technologies, Grand Island, NY, USA). After a second 1 h 4 °C exchange against 10 mM sodium borate buffer (pH 7.1) containing 10 mM β-mercaptoethanol, the reduced CTA1 subunit was used immediately for reconstitution experiments.

### 3.2. Holotoxin Assembly Detected by SPR

To capture CTA2/CTB_5_ on a SPR sensor slide, 75 μL of ganglioside GM1 (Sigma-Aldrich) at a concentration of 3 μg/mL in 200-proof ethanol was added to a gold-plated SPR sensor and allowed to air dry at room temperature. CTA2/CTB_5_ (Sigma-Aldrich) prepared at a concentration of 40 μg/mL in phosphate-buffered saline containing 0.05% Tween 20 (PBST; Medicago, Research Triangle Park, NC, USA) was then added to the GM1-coated sensor in a 75 μL volume and allowed to dry overnight at 4 °C. Although the CTA2/CTB_5_ complex is advertised as CTB by Sigma, it is prepared from the CT holotoxin by removal of CTA1 [40] and contains the CTA2 subunit as confirmed by reactivity with an anti-KDEL antibody [16] (Figure 5a).

To monitor holotoxin re-assembly, the CTA2/CTB_5_ sensor slide was set in a Reichert (Depew, NY, USA) SR7000 SPR refractometer and exposed to a PBST perfusion buffer for 5 min at a flow rate of 41 μL/min (used for all steps) to establish the baseline signal of 0 RIU. The reduced CTA1 subunit was then added at a final concentration of 1 μg/mL to PBST perfusion buffer containing 1 mM β-mercaptoethanol. When indicated, the perfusion buffer also contained 10% glycerol, 100 μM PBA, or was adjusted to pH 6.5.

When indicated, CTA1 was removed from the perfusion buffer and, after a stable equilibrium was reached, replaced with PDI and/or antibody controls. Because PDI reduction is required for its interaction with CTA1 [14,15,16], 10 μg of PDI was exposed to 1 mM GSH for 5 min before perfusion over the reconstituted holotoxin in a total volume of 1 mL PBST containing 1 mM GSH. The anti-PDI antibody (Enzo Life Sciences, Plymouth Meeting, PA, USA) was used at a 1:10,000 dilution, the 35C2 monoclonal anti-CTA1 antibody [41] was used at a 1:500 dilution, and the anti-CTB antibody (Sigma-Aldrich) was used at a 1:15,000 dilution.

### 3.3. Intoxication Assay with Re-Assembled Holotoxin

To generate a reconstituted holotoxin in solution, 1 μg of the free 21 kDa CTA1 subunit was mixed in 200 μL PBS with 3 μg of the ~65 kDa CTA2/CTB_5_ complex. After 2 h at 4 °C, toxin dilutions of 1 and 0.1 nM were added to the serum-free medium of cultured CHO cells for 2 h at 37 °C. Cells were also challenged with 1 and 0.1 nM of pathogen-produced CT for 2 h at 37 °C. All cells had been seeded to 24-well plates the previous day and were at ~80% confluency when exposed to toxin. Extracts generated from intoxicated and unintoxicated controls cells were processed with an ELISA-based kit (GE Healthcare, Piscataway, NJ, USA) to quantify cAMP levels. Resting levels of cAMP from unintoxicated control cells were background-subtracted from the values obtained from intoxicated cells. The background-subtracted results were then expressed as percentages of the maximal cAMP value obtained from cells exposed to 1 nM of pathogen-produced holotoxin.

## 4. Conclusions

The isolated CTA1 subunit is an unstable protein that assumes a disordered conformation at the physiological temperature of 37 °C. Association with the CT holotoxin maintains CTA1 in a stable conformation which likely protects it from proteolysis during transit through the bacterial periplasm, extracellular medium, and host endomembrane system [9,42,43]. The shift to an unfolded conformation upon holotoxin disassembly in the ER allows CTA1 to attain a translocation-competent state and to activate the ERAD export mechanism [17,18,19,25,26]. Here, we have demonstrated another role for unfolding of the dissociated CTA1 subunit: it eliminates the ER-localized interaction between free CTA1 and CTA2/CTB_5_, thus preventing the non-productive reassembly of a CT holotoxin.
